# Radiological surveillance of formerly asbestos-exposed power industry workers: rates and risk factors of benign changes on chest X-ray and MDCT

**DOI:** 10.1186/1745-6673-9-18

**Published:** 2014-04-29

**Authors:** Christian Eisenhawer, Michael K Felten, Miriam Tamm, Marco Das, Thomas Kraus

**Affiliations:** 1 Institute of Occupational and Social Medicine, RWTH Aachen University, Aachen, Germany; 2 Department of Medical Statistics, RWTH Aachen University, Aachen, Germany; 3 Department of Radiology, Maastricht University Medical Center, Maastricht, The Netherlands; 4 GROW, School for Oncology & Developmental Biology, Maastricht University Medical Center, Maastricht, The Netherlands; 5 Department of Diagnostic Radiology, RWTH Aachen University, Aachen, Germany

**Keywords:** Asbestos, Power industry, Chest X-ray, Early detection, MDCT

## Abstract

**Background:**

To determine the prevalence of asbestos-related changes on chest X-ray (CXR) and low-dose multidetector-row CT (MDCT) of the thorax in a cohort of formerly asbestos-exposed power industry workers and to assess the importance of common risk factors associated with specific radiological changes.

**Methods:**

To assess the influence of selected risk factors (age, time since first exposure, exposure duration, cumulative exposure and pack years) on typical asbestos-related radiographic changes, we employed multiple logistic regression and receiver operating characteristic (ROC) analysis.

**Results:**

On CXR, pleural changes and asbestosis were strongly associated with age, years since first exposure and exposure duration. The MDCT results showed an association between asbestosis and age and between plaques and exposure duration, years since first exposure and cumulative exposure. Parenchymal changes on CXR and MDCT, and diffuse pleural thickening on CXR were both associated with smoking. Using a cut-off of 55 years for age, 17 years for exposure duration and 28 years for latency, benign radiological changes in the cohort with CXR could be predicted with a sensitivity of 82.0% for all of the three variables and a specificity of 47.4%, 39.0% and 40.6%, respectively.

**Conclusions:**

Participants aged 55 years and older and those with an asbestos exposure of at least 17 years or 28 years since first exposure should be seen as having an increased risk of abnormal radiological findings. For implementing a more focused approach the routine use of low-dose MDCT rather than CXR at least for initial examinations would be justified.

## Background

Asbestos is a naturally deposited fibrous mineral that was extensively mined and industrially used over many decades in the 20th century. Today the use of asbestos is largely restricted or banned in many industrialised countries because of well-known adverse health effects. Apart from the carcinogenic effect (lung cancer, laryngeal cancer and malignant pleural mesothelioma), exposure to asbestos dust can cause various benign changes of the lung (asbestosis) and the pleura (pleural plaques and diffuse pleural thickening) [1-5].

Statutory accident insurance institutions in Germany are legally required to actively detect clinical symptoms and conditions depending on occupational history, and to compensate asbestos-related diseases of lung, pleura and larynx. In the 1950s, the International Labour Organization (ILO) introduced an international classification of radiographs standardizing chest X-ray (CXR) reading of patients with pneumoconiosis [6-10]. Since the 1970s, the insurance institutions have therefore organised standardised surveillance programmes for the early detection of asbestos-related diseases [4]. Already at that time, it was accepted that computed tomography had a higher sensitivity and specificity for the early detection of asbestos related changes of lung and pleura, although the required higher radiation dose in former times precluded a wider use in early detection programmes [11]. In spite of the rapidly declining industrial consumption of asbestos in Germany since the beginning of the 1980s, the number of patients at risk of asbestos-induced abnormal radiological findings continued to rise. The main reason for this was the long latency period of up to 40 years or more between first exposure and onset of disease, in combination with a still increasing asbestos consumption 40 years ago.

Reliable indicators of asbestos-induced benign parenchymal and pleural changes would be a very useful planning tool for setting up effective and affordable early detection programmes. Although CT technology has become more and more sensitive with smaller amounts of radiation used, early surveillance programmes in Germany are still generally based on conventional CXR [12-26]. Several reports on the use of MDCTs for early detection are available [27-33]. There is general agreement in the literature that the main factors influencing asbestos-related radiological changes are “age at examination” [13,26,27], “time since first exposure” (TSFE) [12,17-20,24,26,28,30,33], “years of exposure” [13,24,27,33], “cumulative asbestos exposure” [13,15,17,19,22,25,27,31], [33], and “smoking habits” [14,24]. High levels of cumulative exposure and history of smoking were usually shown to be associated with high rates of parenchymal changes. Time dependent factors such as age, TSFE and years of exposure were usually associated with pleural changes. In some studies cumulative exposure has been found to be associated with pleural plaques [34].

Thus the first aim of our study was to determine the prevalence of asbestos-related changes in a cohort of formerly asbestos-exposed power industry workers in two subgroups with different risk profiles using CXR (lower risk group) and MDCT (higher risk group). Our second aim was to assess the importance of common risk factors associated with specific radiological changes such as parenchymal fibrosis and pleural thickening. We did this with the view that determining rates of radiological changes typical for specific risk profiles would help differentiate large scale surveillance and early detection programmes.

## Methods

### Cohort selection

Enrolment for the survey was originally started in the late 1990s as a company internal programme of a major provider of electrical power in Germany. Main objective of this programme was the registration of asbestos-exposed active and former employees. All persons were included in the cohort who could be contacted and who replied with a signed statement that they had been exposed to asbestos fibres. The individual cumulative exposure was determined by self-reported job titles and main occupational tasks. These data were analysed in a computer programme developed by occupational and safety experts and based on ambient monitoring data of airborne asbestos fibre concentrations at defined workplaces and the typical occupational tasks and time periods [35]. The cumulative asbestos exposure (in fibres/cubic centimetre × years) was expressed as the product of the eight-hour time weighted average fibre concentration and the total duration of exposure. One fibre year was defined as an exposure during 1920 work hours through daily eight-hour shifts over 240 workdays spread over 48 weeks with a standard airborne concentration of one fibre per cubic centimetre or 1 × 10^6^ fibres per cubic metre.

Of the 5622 individuals who submitted a statement on asbestos exposure, we examined 4446 (79%) during the study period.

The remaining 1176 were not examined for various reasons: contact too late or data incomplete: 44%, refusal to participate: 21%, appointment cancelled: 6%, acute life-threatening disease: 0.6%, deceased before intended examination: 12%, no reply: 5%, personal reasons: 7% and others: 4.4% [36]. Another 42 examined participants had no radiological results because they refused any X-ray examination, or results of recently performed external examinations were not communicated. Of the remaining 4404 participants with valid CXR or CT results, we included only those with a complete set of data (n = 3257, 58% of the total cohort, CXR n = 3061, MDCT n = 196) in the analysis. All participants were former or still active workers of a major provider of electrical power in Germany, most of them stationed in one of six power plants fired with lignite or coal. Most workers had been stationed in more than one plant during their career. Job characteristics were available for 94% (n = 5284) of the total cohort indicating that 30% of them were metalworkers including welders, insulators and mechanics. Another 13% were electricians, including communication technicians, and 30% plant operators, including system controllers and boiler operators. 11% were employed as other craftsmen such as rooters, pipefitters, car mechanics, masons, gauge mechanics, turners, smelters, fire fighters, drivers, painters, warehousemen and forklift operators. A further 4% were present in the contaminated area but not conducting manual work for example work planners. 12% had other occupations such as security personnel, office staff or receptionists. More than half of the workers with data (59% of 5284) were regularly and actively involved in revisions of steam turbines, 21% in removal of asbestos lagging and 4% in spraying of asbestos pulp. Another 36% were involved in tooling and handling of asbestos-containing gaskets, 30% in tooling packings, 26% in removal of asbestos mats, 28% in tooling gauges and 15% in maintenance of heat resistant wires [35]. Between March 2002 and the end of 2006 when the survey was closed for new admissions we invited the participants for standard medical examinations, including radiological examination of the thorax with CXR or MDCT.

Routine surveillance examinations were done in accordance with regulations by the German Social Accident Insurance (DGUV). Based on exposure history, smoking history and age at the time of examination, a subgroup of participants was considered as having an increased risk of developing asbestos-related radiologic changes. These participants were examined with MDCT [37]. CXR was not done in this subgroup. The rest of the cohort was examined by CXR (posterior anterior only). To classify the MDCT group, an empiric multiplicative risk calculation was used combining the factors exposure duration (years), (age/50)^3^ and smoking history (non-smoker = 0.1, ex-smoker = 0.3, smoker = 1.0). The resulting score was seen as a measure of the individual risk of developing asbestos-related radiological changes. A minimum score of 35 was required for admission to the MDCT subgroup.

The research obtained approval by the Institutional Review Board of the Medical Faculty, RWTH Aachen University (registration number EK2205).

### Screening protocol for MDCT

In our study, MDCT examination of the whole lung in supine position during one breath-hold with deep inspiration without administration of contrast material was applied (SOMATOM Sensation 16, Siemens Medical Solutions, Forchheim, Germany). A standard low-dose MDCT protocol was used: 120 kV, 10 mAs_eff_ for individuals with less than 80 kg, 20 mAs_eff_ for individuals with 80 kg and more, 16 × 0,75 mm collimation, rotation time 0,5 seconds, table feed/rotation 18 mm. Images were reconstructed in three different ways. The first stack of images was reconstructed with 5 mm effective slice thickness applying an increment of 4 mm with a medium smooth soft tissue convolution kernel (Siemens B30 kernel) window setting (center C = 80 HU, window W = 400 HU) for analysis of soft tissue changes, mediastinal changes and pleural changes. The next stack of images was reconstructed as 1 mm thick sections with a reconstruction increment of 0.5 mm and a sharp kernel (Siemens B50 kernel) (C = -600; W = 1500) for detection of pulmonary nodules, and the last stack of images was reconstructed as a high-resolution set with 1 mm thick sections every 10 mm with a B80 ultra-sharp reconstruction kernel (C = -600; W = 1500) for analysis of additional asbestos-related changes [37].

### Diagnosis of asbestosis and pleural changes on CXR and MDCT

CXR was read according to the ILO 2000 classification [10]. A small opacity profusion of grade 1/1 (or higher) at least in both lower fields was considered as a sign of asbestosis. Asbestos-related pleural change was defined as circumscribed (local) pleural thickening irrespective of location or dimension, or diffuse pleural thickening of grade “2a” (or higher) at least in both middle and lower fields (according to the ILO classification). MDCT reading was based on the international CT-classification for pneumoconiosis, which has a similar format as the ILO classification for CXR reading [9,38,39]. We considered the following to be a sign of asbestosis on MDCT: bilateral interlobular septal thickening or intralobular lines (grade one or higher) at least in the lower fields, or parietal or visceral pleural changes irrespective of location and dimension.

The changes on CXR were basically grouped in those indicating any changes (parenchymal or pleural), asbestos-related pulmonary fibrosis with or without pleural lesions (asbestosis) and plaques or diffuse pleural thickening with or without asbestosis. The reading results of the MDCTs were grouped in any changes (parenchymal or pleural), asbestosis with or without pleural lesions and pleural thickening (plaques) with or without asbestosis, including parietal and visceral changes (Table 1) [10,40].

**Table 1:** Number of participants with specific changes on standard chest X-ray (n=3061) and MDCT of the thorax (n=196)

		**n**	**%**
	Chest X-ray		
Any changes		422	13.8
Asbestosis		32	1.0
Plaques		242	7.9
Diffuse pleural thickening		180	5.9
No changes		2639	86.2
	MDCT		
Any changes		118	60.2
Asbestosis		40	20.4
Plaques		112	57.1
No changes		78	39.8

CXR and MDCT were read by two independent experienced readers. In cases of disagreement, a consensus reading was required. All readers were specialists in thoracic imaging, radiologists or specialists in occupational medicine.

### Statistical analysis

For continuous variables mean and standard deviation as well as median, minimum and maximum are given. Categorical data are presented with frequencies and percentages.

Multiple logistic regression analysis was used to investigate the association of radiological changes with age, TSFE, years of exposure, cumulative exposure and pack years. Because of strong collinearities among the independent variables age (VIF = 3.0 for the cohort with CXR, VIF = 1.3 for the cohort with MDCT), TSFE (VIF = 8.2 for the cohort with CXR, VIF = 4.2 for the cohort with MDCT) and years of exposure (variance inflation factor [VIF] = 6.6 for the cohort with CXR, VIF = 3.8 for the cohort with MDCT), separate models were fitted including burden of smoking (pack years), cumulative exposure (fibre years) and one of the three collinear variables. For the analysis of the fibre year values, we used three categories considering those with a calculated cumulative asbestos burden of less than or equal to one fibre year as negligibly exposed, as they had often been in contact with asbestos for only a few weeks. Because a fibre year value of 25 and more was a precondition for compensation payments in the German litigation systemfor lung cancer, we defined this as a cut-off value. We considered participants falling in the medium category with values between more than one and less than 25 years as notably exposed, but carrying only a limited additional asbestos-related risk of disease.

In order to determine the expected yield of health surveys among high-risk individuals for detecting asbestos-related abnormal radiological findings, we calculated receiver operating characteristic (ROC) curves. The ROC curves were used to determine the optimal cutpoint for age, TSFE and years of exposure to predict benign radiological changes when using standard examination with CXR. For the high-risk group with MDCT no cutpoints are given because the ROC curves yielded no acceptable discrimination (in all cases the area under the curve (AUC) does not exceed 0.6). To achieve an acceptable sensitivity, we decided to keep the sensitivity fixed in such a way that we attained a minimum sensitivity of 80% and maximized the specifity over the set of eligible cut-off values [41].

Particulary due to the large number of missing fibre year values for the variable cumulative asbestos exposure, the number of observations which could be used in the multiple logistic regression model was reduced to n = 3061 for the subgroup with CXR examination and to n = 196 for that with MDCT. To account for this fact, sensitivity analyses was done fitting the multiple regression models without the variable cumulative exposure so that n = 4158 observations for the cohort with CXR examination and n = 202 observations for the cohort with MDCT were taken into account.

All applied tests were two-sided. Statistical analyses were performed in an explorative manner; thus p-values of p ≤ 0.05 could be interpreted as statistical significant test results.

ROC curves were calculated using the software MedCalc Version 11.4.4.0, Mariakerke, Belgium. All other analyses were carried out with the statistical software SAS Version 9.2, SAS Institute Inc., Cary, North Carolina, USA.

## Results

### Prevalence of asbestos-related radiological changes

The CXR group was characterised by low rates of both asbestos-related pulmonary changes (1%) and pleural changes (plaques 7.9%, diffuse pleural thickening 5.9%) when compared to the corresponding rates found in the MDCT-group (20.4% and 57.1%). Table 1 shows the number of participants in the CXR and MDCT-groups with radiological changes typical for benign asbestos-related findings of lung and pleura. While less than 15% of the CXR-group showed abnormal radiological findings, two thirds (60.2%) of the CT-group had typical changes. We explained the difference with the fact that the CT group had been preselected on the basis of a higher asbestos burden, and a correspondingly higher risk of disease, and the better performance of the MDCTs.

In the CXR group, we found only 32 cases of asbestos-related changes of the lung (asbestosis), but a much higher rate of plaques (n = 242) and diffuse pleural thickening (n = 180). It was remarkable that the prevalence of diffuse pleural thickening and plaques was with 5.9% and 7.9% quite similar. In the CT-group, the predominance of plaques (n = 112) was less pronounced with an only three-fold rate, possibly due to higher rates of prevalence and detection.

### Importance of risk factors

Table 2 shows the prevalence of the five applied risk factors stratified by method of investigation (CXR or MDCT). As expected from the process of allocation to the two groups, the CT group had higher mean values for age, TSFE, years of exposure, cumulative exposure and pack years. Individuals with a high asbestos burden and those with only a minor exposure could be found in both subgroups.

**Table 2:** Prevalence of applied risk factors stratified by method of investigation (chest X-ray or MDCT)

	**Mean**	**Std. Dev.**	**Median**	**Minimum**	**Maximum**
**Chest X-ray**					
Age (years)	57.0	11.5	58.0	27.0	90.0
TSFE^a^ (years)	32.4	10.9	31.0	3.0	65.0
Exposure duration (years)	21.3	10.1	20.0	<1	53.0
Cumulative exposure (fibre years)	42.2	129.5	4.9	<1	2331.4
Smoking (pack years)	19.7	22.5	14.0	0	136.5
**Low-dose MDCT**					
Age (years)	65.8	6.1	65.5	41.0	84.0
TSFE^a^ (years)	41.2	7.4	41.0	20.0	57.0
Exposure duration (years)	29.8	7.5	30.0	4.0	46.0
Cumulative exposure (fibre years)	78.6	216.6	18.0	<1	2558.2
Smoking (pack years)	43.2	27.9	41.0	0	118

^a^time since first exposure.

The multiple logistic regression analysis of the CXR results indicated a significant effect of all considered risk factors (cumulative exposure only when comparing the groups with ≤1 and ≥ 25 fibre years) on the outcome changes. Signs of asbestosis on CXR were associated with all risk factors. However, we found an inverted effect of fibre year values on asbestosis (Table 3). The presence of plaques was mainly associated with age, TSFE and exposure duration. After adjusting for age, some effect could also be seen for cumulative exposure, especially when comparing the groups with less than or equal to one and 25 or more fibre years. Diffuse pleural thickening was associated with all risk factors including pack years, except cumulative exposure. When defining the variables age, TSFE and exposure duration as mainly time related and considering the variable cumulative exposure primarily as asbestos dose related, we can say that on CXR signs of asbestosis and diffuse pleural thickening were primarily associated with time whereas plaques were associated with both, time and exposure.

**Table 3:** Changes on standard chest X-ray: results of multiple logistic regression model (n = 3061, odds ratios with 95% confidence interval and p-values of Wald test)

		**Age**	**TSFE**^**f**^	**exposure duration**	**Cumulative exposure (fibre years)**		**Pack years**
		**(years)**	**(years)**	**(years)**	**>1 & <25 vs. ≤ 1**^**b**^**≥25 vs. ≤1**^**c**^		
	**Model**^**a**^	**OR**^**d**^**/ CI**	**OR**^**d**^**/ CI**	**OR**^**d**^**/ CI**	**OR / CI**	**OR / CI**	**OR**^**d**^**/ CI**
		**p**	**p**	**p**	**p**	**p**	**p**
**Any changes**	m1	1.86 / 1.67-2.07	-	-	1.23 / 0.93-1.61	1.51 / 1.12-2.04	1.06 / 1.01-1.10
**n = 422 (13.8 %)**		<0.0001			0.1433	0.0069	0.0138
	m2	-	1.68 / 1.51-1.86	-	1.19 / 0.90-1.55	1.35 / 0.99-1.83	1.07 / 1.02-1.11
			<0.0001		0.2183	0.0505	0.0045
	m3	-	-	1.71 / 1.53-1.92	1.17 / 0.89-1.53	1.29 / 0.95-1.75	1.06 / 1.02-1.11
				<0.0001	0.2554	0.1048	0.0071
**Asbestosis**	m1	3.22 / 2.11-4.92	-	-	0.32 / 0.12-0.82^e^	1.20 / 0.53-2.70	1.16 / 1.02-1.31
**n = 32 (1.0 %)**		<0.0001			0.0186	0.6617	0.0216
	m2	-	2.01 / 1.41-2.86	-	0.31 / 0.12-0.80^e^	0.99 / 0.44-2.27	1.17 / 1.04-1.33
			<0.0001		0.0154	0.9874	0.0125
	m3	-	-	2.13 / 1.45-3.12	0.30 / 0.11-0.78^e^	0.90 / 0.39-2.09	1.16 / 1.03-1.32
				<0.0001	0.0131	0.8042	0.0169
**Plaques**	m1	1.84 / 1.61-2.10	-	-	1.43 / 1.01-2.05	1.64 / 1.11-2.42	1.03 / 0.98-1.09
**n = 242 (7.9 %)**		<0.0001			0.0470	0.0128	0.2536
	m2	-	1.70 / 1.49-1.93	-	1.38 / 0.97-1.97	1.45 / 0.98-2.15	1.04 / 0.99-1.10
			<0.0001		0.0754	0.0659	0.1538
	m3	-	-	1.69 / 1.47-1.95	1.37 / 0.96-1.96	1.40 / 0.94-2.09	1.04 / 0.98-1.10
				<0.0001	0.0821	0.1000	0.1939
**Diffuse pleural thickening**	m1	1.67 / 1.44-1.94	-	-	1.20 / 0.81-1.78	1.42 / 0.93-2.19	1.06 / 1.00-1.13
**n = 180 (5.9 %)**		<0.0001			0.3542	0.1080	0.0482
	m2	-	1.56 / 1.35-1.81	-	1.17 / 0.79-1.73	1.29 / 0.83-2.00	1.07 / 1.01-1.14
			<0.0001		0.4396	0.2600	0.0267
	m3	-	-	1.60 / 1.36-1.89	1.15 / 0.78-1.71	1.23 / 0.79-1.91	1.07 / 1.01-1.14
				<0.0001	0.4830	0.3717	0.0344

^a^m1 = model with age, m2 = model with latency, m3 = model with years of exposure.

^b^group with >1 and < 25 fibre years versus group with ≤1 fibre years.

^c^group with ≥25 fibre years versus group with ≤1 fibre years.

^d^odds ratio for an increase of 10 years (continuous variables).

^e^results not plausible.

^f^time since first exposure.

In the CT-group, the outcome variable of any changes was associated with TSFE and the cumulative exposure (considering the model adjusting for age). Signs of asbestosis were associated only with age and pack years, whereas pleural plaques were associated with TSFE, exposure duration and fibre years (group with ≤1 versus ≥ 25 fibre years, considering the model adjusting for age) (Table 4). Consistent with the CXR results, signs of asbestosis were only time-related and pleural lesions (plaques) were both time- and dose- related.

**Table 4:** Changes on MDCT: results of multiple logistic regression model (n = 196, odds ratios with 95% confidence interval, p-values of Wald test)

		**Age**	**TSFE**^**e**^	**Exposure duration**	**Cumulative exposure (fibre years)**		**Pack years**
		**(years)**	**(years)**	**(years)**	**>1 & <25 vs. ≤ 1**^**b**^**≥25 vs. ≤1**^**c**^		
	**Model**^**a**^	**OR**^**d**^**/ CI**	**OR**^**d**^**/ CI**	**OR**^**d**^**/ CI**	**OR / CI**	**OR / CI**	**OR**^**d**^**/ CI**
		**p**	**p**	**p**	**p**	**p**	**p**
**Any changes**	m1	1.53 / 0.94-2.50	-	-	1.99 / 0.87-4.51	2.54 / 1.12-5.76	1.06 / 0.95-1.18
**n = 118 (60.2 %)**		0.0885			0.1015	0.0255	0.3024
	m2	-	1.54 / 1.02-2.31	-	1.94 / 0.85-4.43	2.12 / 0.92-4.89	1.06 / 0.95-1.18
			0.0398		0.1149	0.0779	0.2980
	m3	-	-	1.47 / 0.98-2.22	2.00 / 0.88-4.56	2.13 / 0.92-4.92	1.05 / 0.94-1.17
				0.0636	0.0980	0.0768	0.3736
**Asbestosis**	m1	2.13 / 1.11-4.07	-	-	1.68 / 0.56-5.07	1.27 / 0.41-3.91	1.15 / 1.02-1.30
**n = 40 (20.4 %)**		0.0228			0.3594	0.6808	0.0244
	m2	-	1.58 / 0.95-2.62	-	1.68 / 0.56-5.06	1.06 / 0.34-3.31	1.14 / 1.01-1.29
			0.0784		0.3546	0.9207	0.0343
	m3	-	-	1.09 / 0.66-1.81	1.85 / 0.62-5.52	1.25 / 0.40-3.90	1.14 / 1.01-1.28
				0.7257	0.2675	0.6998	0.0394
**Plaques**	m1	1.52 / 0.93-2.47	-	-	1.45 / 0.64-3.28	2.54 / 1.12-5.76	1.05 / 0.95-1.17
**n = 112 (57.1 %)**		0.0939			0.3724	0.0253	0.3491
	m2	-	1.61 / 1.07-2.41	-	1.40 / 0.62-3.19	2.09 / 0.90-4.82	1.05 / 0.95-1.17
			0.0225		0.4235	0.0847	0.3440
	m3	-	-	1.55 / 1.03-2.33	1.45 / 0.64-3.29	2.09 / 0.90-4.83	1.04 / 0.94-1.16
				0.0368	0.3752	0.0846	0.4329

^a^m1 = model with age, m2 = model with latency, m3 = model with years of exposure.

^b^group with >1 and < 25 fibre years versus group with **≤**1 fibre years.

^c^group with ≥ 25fibre years versus group with ≤ 1 fibre years.

^d^odds ratio for an increase of 10 years (continuous variables).

^e^time since first exposure.

The results of the sensitivity analyses show that the statistical results in the multiple logistic regression were only marginaly affected by the reduction of number of observations due to the large number of missing values in the variable fibre years. In the CXR group the effect of pack years on diffuse pleural thickening was no longer significant. In the CT group age and years of exposure became significant for any changes and TSFE for asbestosis.

### ROC analysis

Using ROC analysis to predict the outcome “any changes” with a minimum sensitivity of 80% and optimal specificity, we calculated in the CXR group cut-off values for age (55 years, area under the curve (AUC) = 0.694), TSFE (28 years, AUC = 0.666) and exposure duration (17 years, AUC = 0.660). With a sensitivity of 82% we obtained specificities of 47%, 41% and 39% for age TSFE and exposure duration. For the CT group we achieved no acceptable discrimination to develop good cut points (AUC < 0.6), possibly due to the small sample size and the selection criteria applied for this group.

## Discussion

Our cohort of 3257 asbestos-exposed workers had started their careers in the power industry when raw asbestos and asbestos-containing materials were routinely handled without effective protection.

The participants had been registered for a surveillance programme primarily designed for early detection of asbestos-related lung cancer. However, in the framework of our evaluation we did not consider cases of malignant asbestos-related diseases, but focused on benign asbestos-related changes only. The type of the initial radiological examination was decided on the estimated risk of developing asbestos-related changes. As the allocation to the group with MDCTs was based on a high risk score (older participants with long exposure duration and extended smoking history), the two groups with MDCT and CXR were different in terms of age, exposure duration and smoking status (Table 2). This together with the different reading routines made it necessary to consider both groups separately. We could still draw conclusions on the key role factors of radiological changes, which were reflected in the findings of both groups.

### Chest X-ray

In CXR-based studies, an association between TSFE and the risk of developing pleural plaques is predominant. Jakobsson found a strong correlation between pleural plaques and TSFE, while the association with the cumulative asbestos exposure was much weaker [17]. The correlation between pleural plaques and TSFE was confirmed by Koskinen, who could also show some association with duration of exposure in a cohort of 18943 workers from construction industry, shipyards and asbestos industry in Finland [18]. Ehrlich found an exclusive correlation to TSFE in a multivariate analysis without any influence of cumulative exposure and exposure duration [19]. In a further study employing multivariate analysis, Matrat described a significant influence of TSFE for developing pleural thickening in 277 custodian and maintenance employees with generally low asbestos exposure [20]. In other studies, combined associations of age [26], duration of exposure, pack years and TSFE [24] were shown. Metintas described for a group of 991 villagers with environmental asbestos exposure in Turkey, an influence of age, gender, duration of exposure and type of asbestos on the development of local pleural thickening, whereas diffuse pleural thickening was influenced by age, duration of exposure and cumulative exposure [13].

Recent studies found that the cumulative exposure is the most important risk factor for the prevalence of asbestosis [15,17,19,21-25]. A correlation with smoking habits has been published [16,42]. In contrast Koskinen found a correlation to TSFE [18], Cookson [12] to TSFE and total exposure and Lilis [26] to TSFE and pack years. In our study, age, TSFE and duration of exposure and less pack years are significant risk factors for the prevalence of asbestosis. The inverse association between asbestosis and fibre year values (OR < 1) did not seem plausible. These results are not corresponding with the most cited studies and may be the result of an effect of dose- related factors on the prevalence of asbestosis. We regard the low prevalence of asbestosis in CXR (n = 32) as one reason (see Table 1). As a second reason, the inverted association between asbestosis and fibre year values in the analysis of the group with ≤1 fibre years versus the group with >1 and <25 fibre years was probably the result of misinterpretation of smoking-related changes in the lower fields as asbestosis. In the group with ≤1 fibre years we found increased pack-year rates compared with the other groups, especially the group with >1 and <25 fibre years.

There was also a significant association between asbestosis and pack years as well as between diffuse pleural thickening and pack years (Table 3). In this regard, it is difficult to distinguish between asbestos and cigarette smoke-related changes on CXR examination in some cases. The statistical analysis of pleural changes in CXR in the largest group of plaques (n = 242) and in the group of diffuse pleural thickening (n = 180) shows a significant influence of age, exposure duration and TSFE as time-based risk factors, in accordance with the predominant literature. We also found a significant influence of cumulative exposure in the group of plaques. This result is not corresponding to the most cited studies. The low rate of asbestosis on CXR (1.0%) and comparatively high rates of pleural changes (plaques 7.9%, diffuse pleural thickening 5.9%) was consistent with other published results (24). The expected predominance of diffuse pleural changes could not be shown, possibly due to the applied strict criteria together with the poor performance of the CXR in differentiating between the two pleural lesions. That may have led to readings of diffuse pleural thickening to be underestimated.

The ROC analysis results in optimal cut-offs of 55 years for age, 17 years for the exposure time and 38 years for latency to discriminate between patients with and without radiological chances. These cut-offs do not achieve an excellent discrimination as presented with the AUC. However, we decided to keep the sensitivity fixed, so that 82% of the patients of our cohort would be found to have radiological changes. It should be noted that the cut-off points are based on the particular cohort selected here for CXR. These would need to be validated in further studies.

### MDCT

Paris noted a significant association between signs of fibrosis on high resolution CT (HRCT) and cumulative asbestos exposure in a cohort of 706 retired workers with a known occupational asbestos exposure in different industrial companies [27]. This study assessed the risk factors age, gender, smoking, duration of exposure and cumulative exposure index. The prevalence of pleural plaques was not examined in this study. In a further study of Paris, TSFE was the key variable for both, pleural plaques and asbestosis in a cohort of 1011 asbestos-exposed subjects in France [28]. Several parameters were calculated (TSFE, duration, intensity and cumulative exposure to asbestos and age). The cumulative exposure was also considered significant.

In a recent study using HRCT, pleural plaques were associated with both TSFE (p < 0.0001) and dose parameters (cumulative exposure (p = 0.02)) [33]. The prevalence of asbestosis was significantly associated only with dose parameters. No relationship was observed between duration of exposure and these two asbestos-related changes. The following risk factors were examined by logistic regression: TSFE, cumulative exposure index and intensity of exposure (adjusted for smoking status and body mass index). Schaeffner described a significant association between cumulative exposure index (level and duration of exposure) and simultaneous presence of pleural plaques and asbestosis [31]. TSFE was the main risk factor for both pleural plaques and asbestosis in a study of Algranti and lower association between asbestosis and cumulative exposure index [30]. In contrast, Cleemput could find no significant association between cumulative exposure or TSFE and the prevalence of pleural plaques [32]. In a recent study of Mastrangelo based on MDCT, the following variables were used in statistical analysis: Age, smoking habits, cumulative exposure, peak asbestos level, length of exposure and TSFE [29]. The results of the logistic regression models with stepwise selection of variables showed a significant association between cumulative exposure and asbestosis and TSFE and peak exposure and pleural plaques. TSFE was also correlated with diffuse pleural thickening.

In the analysis of MDCT, our results show a significant association between plaques, years of exposure and TFSE as time-based risk factors and cumulative exposure as a dose-based risk factor (Table 4). The association between asbestosis and cumulative exposure was described in several cited studies but not in our analysis. However, our cohort for MDCT was quite small with n = 196. For asbestosis, we found a significant association with age as time-based risk factor and pack years. In summary, we could show that time-based and dose-based risk factors were both associated with radiological changes indicating plaques. Parenchymal changes were associated primarily with the time-based factor age and corresponding to the results in our CXR-group with pack years as a sign of probable misinterpretation of smoking-related changes. In contrast to several cited studies, dose-based risk factors such as exposure duration and cumulative exposure were not associated with parenchymal changes. Considering the low threshold we used for diagnosing asbestosis (bilateral interlobular septal thickening or intralobular lines with grade one or higher at least in the lower fields) and the observed significant association with pack years, we can interpret the results as partly indicating misclassification of irregular opacities in low profusion grades. The detected parenchymal changes were probably not a sign of asbestosis but rather nonspecific changes probably related to smoking.

In MDCT examination we found an increased prevalence of asbestosis (n = 40, 20.4%) and plaques (n = 112, 57.1%) compared with CXR. The increased prevalence of asbestosis and pleural changes in MDCT examination can be explained by the higher sensitivity than CXR technology and the selection of this subgroup with the estimated higher duration of asbestos exposure.

## Conclusion

We found a strong association between asbestos-induced radiological changes and the time-related risk factors age, TSFE and exposure duration. There was also a weak association between pleural lesions (CXR and MDCT) and cumulative exposure. Time-related factors had an overriding influence on most considered response variables excluding local plaques, which were also associated with the cumulative exposure. In comparable cohorts of asbestos exposed workers using a risk-dependent cohort selection, similar rates of benign asbestos-related changes of lung or pleura using CXR (14%) or MDCT (60%) can be expected.

In order to focus health surveillance efforts for asbestos-related changes using CXR in asbestos-exposed workers, those aged 55 years and older should be examined with first priority. Alternatively, or after applying as a second criterion, those with an asbestos exposure of at least 17 years or with at least 28 years since first exposure should be screened. A more focused approach with a minimum number of individuals examined per case detected would also justify the routine use of MDCT rather than CXR for initial examination.

## Competing interests

The authors declare that they have no competing interests.

## Authors’ contributions

CE coordinated the examination of participants, extracted the relevant data for analysis, interpreted the results and drafted the manuscript, MKF organized the cohort, managed the survey data and interpreted the results, TK conceived the study, designed the building of the cohort and the framework of the survey and interpreted the results. MT analysed the study data and supported their interpretation. MD evaluated the radiological method and data and revised them critically for important radiological content. All authors read and approved the final manuscript.
